# Magnetic isolation of *Plasmodium falciparum* schizonts iRBCs to generate a high parasitaemia and synchronized *in vitro* culture

**DOI:** 10.1186/1475-2875-13-112

**Published:** 2014-03-21

**Authors:** Lydia Mata-Cantero, Maria J Lafuente, Laura Sanz, Manuel S Rodriguez

**Affiliations:** 1 Medicines Development Campus, Diseases of the Developing World, GlaxoSmithKline, Severo Ochoa 2, Tres Cantos 28760, Madrid, Spain; 2 Proteomics Unit, CICbioGUNE Ed. 801A Parque Tecnológico de Bizkaia, 48160 Derio, Spain; 3 Ubiquitylation and Cancer Molecular Biology, Inbiomed, Mikeletegi 81, 20009 San Sebastian, Spain

**Keywords:** *Plasmodium falciparum*, High parasitaemia, Synchronous cultures, Magnetic columns

## Abstract

**Background:**

The establishment of methods for an *in vitro* continuous culture of *Plasmodium falciparum* is essential for gaining knowledge into its biology and for the development of new treatments. Previously, several techniques have been used to synchronize, enrich and concentrate *P. falciparum*, although obtaining cultures with high parasitaemia continues being a challenging process. Current methods produce high parasitaemia levels of synchronized *P. falciparum* cultures by frequent changes of culture medium or reducing the haematocrit. However, these methods are time consuming and sometimes lead to the loss of synchrony.

**Methods:**

A procedure that combines Percoll and sorbitol treatments, the use of magnetic columns, and the optimization of the *in vitro* culture conditions to reach high parasitaemia levels for synchronized *Plasmodium falciparum* cultures is described.

**Results:**

A new procedure has been established using *P. falciparum* 3D7, combining previous reported methodologies to achieve *in vitro* parasite cultures that reach parasitaemia up to 40% at any intra-erythrocytic stage. High parasitaemia levels are obtained only one day after magnetic column purification without compromising the parasite viability and synchrony.

**Conclusions:**

The described procedure allows obtaining a large scale synchronized parasite culture at a high parasitaemia with less manipulations than other methods previously described.

## Background

Malaria is one of the deadliest infectious diseases in the world. It is responsible for more than 300 million clinical cases and over two million deaths annually [1]. The most severe form is caused by the protozoan parasite *Plasmodium falciparum. Plasmodium* has a complex multistage life cycle, with sexual reproduction in the *Anopheles* mosquito and asexual phase within the human host, where it develops in liver and erythrocytic cells. The establishment of an *in vitro* continuous culture of *P. falciparum* is essential for gaining insight into parasitic immunology, biology and pathogenesis as well as for the development of new drugs and vaccines [2-5]. The intra-erythrocytic stage of the malaria parasite is the primary target for anti-malarial drug development as it is associated with pathogenesis. Therefore, main efforts have been focused on the development of intra-erythrocytic parasite cultures [3]. Although some culture conditions have been improved, the technique is essentially the same as described by Trager and Jensen [4], and difficulties to obtain cultures with a parasitaemia higher than 10% still remain. Theoretically the parasitaemia of an *in vitro P. falciparum* culture could increase up to 16-fold per life cycle, however only an increase of three- to eight-fold is observed in 48 hours [6,7]. The inhibition of malaria parasite development has been associated with medium acidification due to the secretion of lactic acid [8,9]. Other mechanisms have also been proposed to explain the regulation of parasite density including increased parasite apoptosis [10] through a quorum sensing mechanism [11]. This process is based on the production of low molecular mass-signalling molecules called auto-inducers, which serve as a protective mechanism by restricting parasite propagation [10,11].

Proteomic studies [12] can contribute to understanding the biology of this complex parasite and also to identifying potential drug and vaccine targets. However, uninfected red blood cells (uRBCs) can interfere in functional proteome analysis of the malaria intra-erythrocytic cycle. In this regard, there is a real need for the achievement of a synchronized *P. falciparum* parasite culture with high parasitaemia. Synchronization of *P. falciparum* facilitates the identification of stage-specific proteins while high parasitaemia levels are critical to enrich *P. falciparum* and infected red blood cells (iRBCs) protein content.

*Plasmodium falciparum* tends to grow in synchrony within the human host. This parasite coordination has been linked to change in temperature and circadian rhythms of the human body [13,14], but the factors which produce synchrony in humans are not present *in vitro*. Although several techniques are frequently used to enrich specific asexual stages, the sorbitol method described by Lambros and Vanderberg [15] is the most widely used because it is versatile and easy to perform. This method is based on the differential sugar and anion permeability of infected cells during intra-erythrocytic development. This selective permeability leads to a hypotonic lysis of erythrocytes infected with larger stage parasites, enriching the *P. falciparum* culture with a ring-stage population and uRBCs. Percoll [16] and Percoll-sorbitol gradients [17] in different proportions are other alternatives used to increase synchrony. Ring-stage iRBCs and uRBCs are separated from mature iRBCs after a high-speed centrifugation because mature parasitized iRBCs have lower density. These methods are also widely used to enrich *P. falciparum* cultures with mature iRBCs and to perform invasion assays [18,19].

Another described method to separate and concentrate mature forms from ring-stage iRBCs with a greater efficacy makes use of magnetic columns [20]. This method takes advantage of the presence of haemozoin which is produced by the breakdown of Fe (II)-containing haemoglobin in mature parasitized erythrocytes as it is a significant source of nutrients for the parasite [21-23]. Further studies have improved the methodology using a high magnetic field gradient [23], allowing to synchronize and concentrate mature parasitized iRBCs with a purity higher than 90%, even for those *P. falciparum* strains that do not exhibit knobs [24-27]. This method is particularly suitable for molecular and biochemical analysis of the biology of the parasites, as the viability and morphology of the parasites and RBCs are not affected [28-30]. Moreover, parasites isolated using magnetic columns are able to invade new RBCs in a more reproducible manner than observed for parasites purified with Percoll-sorbitol treatments [29,30].

Current methods to obtain *P. falciparum* cultures with high parasitaemia are based on the daily replacement of culture medium or on culture dilution to low haematocrits [31-33] to preserve parasite viability. This is because the high metabolism rate of the parasite leads to the accumulation of large amount of metabolic products. Continuous flow methods are used to achieve *P. falciparum* cultures at high parasitaemia, but they are expensive and it is not always possible to work with synchronous cultures [5]. Radfar *et al.* established a protocol to produce 50% parasitaemia *P. falciparum* cultures in low haematocrit conditions [33]. This protocol, combined with alternation of sorbitol and 70% Percoll treatments, results in a highly synchronized *P. falciparum* culture. The authors provide an equation to calculate the volume of culture medium required at a given developmental stage of the parasite for a desired parasitaemia in a specified concentration of RBCs [33]. The main disadvantage of the previously described methods is the labour-intensive culturing, as culture medium changes are required at any time for at least two weeks. In addition, it is difficult to avoid parasite stress and to maintain culture synchrony for such a long period.

The protocol proposed here makes use of Percoll-sorbitol treatments together with magnetic column purification to get highly synchronized cultures. This is combined with a reduction in the haematocrit to increase invasion rates, which allows the achievement of high parasitaemia levels of synchronous cultures. Haematocrit and parasitaemia conditions have been established using the *P. falciparum* 3D7 strain. Parasitaemia levels up to 40% are obtained once the iRBCs, isolated by magnetic column purification, invade fresh uRBCs. Furthermore, this method provides a faster, more reproducible and less laborious methodology than previously reported methods to achieve high parasitaemia levels of synchronized parasite in *in vitro* cultures.

## Methods

### *Plasmodium falciparum* culture

The *P. falciparum* strain 3D7A used in this study was obtained from the Malaria Research and Reference Reagent Resource [34]. RBCs were obtained from the Spanish Red Cross Blood Bank. *Plasmodium falciparum* strain 3D7 was grown according to the method previously described by Trager and Jensen [4]. *Plasmodium falciparum* cultures were maintained at 37°C in RPMI 1640 medium (Gibco) supplemented with 5% AlbuMAX II (Invitrogen), and 150 μM hypoxanthine (Sigma-Aldrich) (complete medium) in a 5% CO_2_, 90% N_2_ and 5% O_2_ atmosphere using fresh uRBCs at 1% haematocrit. The parasites were cultured under these conditions in 150 sq cm culture flasks with a maximal culture media volume per flask of 150 ml. Parasitaemia levels were monitored by Giemsa-stained smears of the cultures.

### Synchronization protocol

*Plasmodium falciparum* cultures maintained at 1% haematocrit and at least 2% parasitaemia were centrifuged at 600 g and supernatant was discarded. Twenty volumes (1 V = RBCs pellet volume) of a 5% wt/v sorbitol (sigma) solution were added to the culture pellet and cellular suspensions were incubated with gentle shaking for 10 min at 37°C. After this incubation a new centrifugation step was performed and cellular pellets were washed three times with complete medium. Finally, parasites were resuspended in 100 ml of complete medium at 0.8% haematocrit and 2% parasitaemia and grown for three days. Then, a new cycle of synchronization was performed by Percoll (sigma) treatment according to the protocol described by Radfar *et al*. [33]. In order to obtain a tightly synchronized *P. falciparum* culture, synchronization was carried out when new merozoites were invading new RBCs [33], before all the schizonts had disappeared and young rings were visible. *Plasmodium falciparum* cultures were centrifuged and each 1 ml iRBC pellet was carefully placed on top of 3 ml of 70% Percoll gradient (v/v) (see [33] for preparation). Each tube was centrifuged at 800 g for 10 min and it was stopped with deceleration 0. The top layer containing the schizonts was recovered and washed two times with complete media. Approximately 50 μl of schizonts iRBCs were obtained for each 100 ml of 1% haematocrit *P. falciparum* cultures (around 90% parasitaemia). Each aliquot of purified parasites were maintained in culture for 48 hours using 1 ml of fresh uRBCs in 150 sq cm culture flasks with 100 ml of complete medium (parasitaemia around 5%).

### Magnetic column separation and invasion assays

The most commonly used magnetic cell fractionation system is the commercially available MACS system (Miltenyi Biotec). MACS separation columns ¨CS¨ were placed into the vario MACS® magnetic support and equilibrated by adding 60 ml of pre-warmed (37°C) RPMI medium without supplementation (incomplete medium). Magnetic separation of mature parasite forms from iRBCs was conducted 48 hours after Percoll treatment. Parasite cultures from each 150 sq cm culture flask (100 ml at 1% haematocrit) were centrifuged at 600 *g* for 5 min and then resuspended with 10 ml of incomplete medium. Ten ml of *P. falciparum* culture at 10% haematocrit were loaded on the top of the column. A low flow rate was used to pass the culture through the column. The effluent containing the uRBCs, ring and young trophozoites iRBCs was discarded. Columns were washed using 30 ml of pre-warmed incomplete medium at medium flow. Column was removed from the magnetic field and 30 ml of pre-warmed (37°C) complete medium was added to elute the mature forms. Purified iRBCs recovered from column eluent were counted using a Neubauer chamber and haematocrit and parasitaemia were adjusted with fresh uRBC and complete medium according to the experiment. Before harvesting, *P. falciparum* cultures were washed twice with cold PBS. The number of RBCs was determined by counting with the Neubauer chamber and parasitaemia was estimated by microscopic examination of Giemsa-stained smears.

### *Plasmodium falciparum* growth inhibition assay

The sensitivity of *P. falciparum*-infected erythrocytes to various drugs was determined using the previously described method based on ^3^H-hypoxanthine incorporation assay [35]. Inoculums of 0.5% parasitaemia (ring stage) and 2% haematocrit were prepared from two different *P. falciparum* cultures, both were treated with Percoll and sorbitol, but one of them was subjected to an additional purification step by magnetic columns. The parasites were grown in RPMI 1640, 25 mM HEPES and supplemented with 5% Albumax. Plates were incubated at 37°C, 5% CO_2_, 5% O_2_, and 90% N_2_. After 24 hours of incubation, [3H] hypoxanthine was added and plates (Costar #3894) were incubated for another 24 hours. After that period, plates were frozen and then harvested on a glass-fibre filter using a TOMTEC Cell harvester 96. Filters were dried and melted on scintillator sheets and the bound radioactivity was quantified by use of a Wallac Microbeta Trilux (Model 1450 LS- Perkin Elmer). IC50s were determined using GraFit 5 (Erithacus Software, Horley, Surrey, UK).

## Results

### Synchronization and purification of *Plasmodium falciparum* 3D7 cultures

The 3D7 *P. falciparum* strain, commonly utilized for *in vitro* anti-malarial studies, was used for the development of the methodology. *P. falciparum* cultures were maintained at low haematocrit conditions (0.8-1%) to scale up the culture fastly as it has been previously reported that haematocrit has an influence on the parasite invasion rate. Parasite cultures easily reached 5-15% of parasitaemia with a media change of three times per week and the addition of new uRBC when parasites were in mature stages to facilite the invasion. A synchronization process by sorbitol treatment was carried out to enrich ring-stage parasites. *Plasmodium falciparum* culture was maintained at 0.8% haematocrit and 2% parasitaemia. After 72 hours a new synchronization was done with 70% Percoll to enrich mature-stage population. Both synchronization treatments were alternated to avoid toxicity and to obtain a good synchrony window before performing the magnetic column purification. Results were optimal if the synchronization treatments were carried out when the schizonts were invading new RBCs and early rings were present in culture.

**Figure 1:**
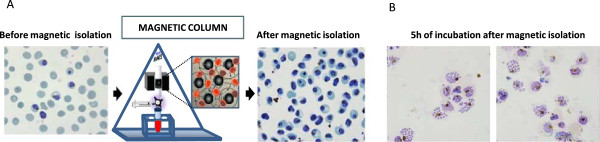
**Isolation of schizonts enriched *Plasmodium falciparum *infected red blood cells with vario mac magnetic columns. A.** Culture of mature forms of *P. falciparum* iRBC previously synchronized with sorbitol and Percoll at haematocrit 1 and P = 10% (blood smear left panel). iRBCs were passed though the magnetic column. Giemsa blood smear of the right panel shows the iRBCs obtained after the purification. **B.** Blood smears of iRBCs purified with the magnetic column after five hours of incubation. Merozoites and schizonts releasing merozoites can be observed.

Forty-eight hours after Percoll treatment, cultures in schizont stage and a parasitaemia around 10% were subjected to magnetic column purification. Only mature parasitized RBCs were retained by the magnetic field and a highly pure parasite population of iRBCs at 98% parasitaemia was eluted from the column, once magnetic field was removed (Figure 1). Table 1 summarizes the total number of iRBCs purified with magnetic columns, starting with a synchronized culture of 100 ml at 1% haematocrit and around 10% parasitaemia. This protocol was very reproducible and approximately 1 × 10^8^ iRBCs were obtained by purification. If a higher amount of iRBCs is required, scale up is possible.

**Table 1:** **Number of infected red blood cells purified, starting from 100 ml of culture H = 1**% **and P = 10**% **(n = 8)**

**No**	**iRBCs after MACs purification**
1	9.63 × 10^7^
2	1.21 × 10^8^
3	8.50 × 10^7^
4	8.40 × 10^7^
5	1.10 × 10^8^
6	6.95 × 10^7^
7	8.33 × 10^7^
8	2.75 × 10^8^
Average	1.16 × 10^8^ ± 0.67 × 10^7^

To verify the ability of iRBC purified by magnetic columns to progress along the life cycle, *P. falciparum* cultures were examined by microscopy using Giemsa stained smears five hours after purification (Figure 1B). Figure 1 shows that the number of merozoites and schizonts releasing new merozoites was significantly higher than 60% in all the cases. These merozoites can be purified through a 1.2 μm Acrodisc 32-mm syringe filter (Pall) and be used for invasion studies [36]. This achievement is relevant as production of merozoites is critical for a better understanding of the parasite invasion process and available protocols to isolate merozoites are not very efficient. The level of synchrony of cultures is essential to achieve enriched-merozoite populations.

### Establishment of the conditions to obtain *Plasmodium falciparum* cultures at high parasitaemia after magnetic column purification

High parasitaemia can be achieved by decreasing the haematocrit or with frequent media replacements. In order to obtain the highest invasion rates and the maximum parasitaemia after the magnetic column purification, several studies were conducted to determine the suitable haematocrit and initial parasitaemia.

**Figure 2:**
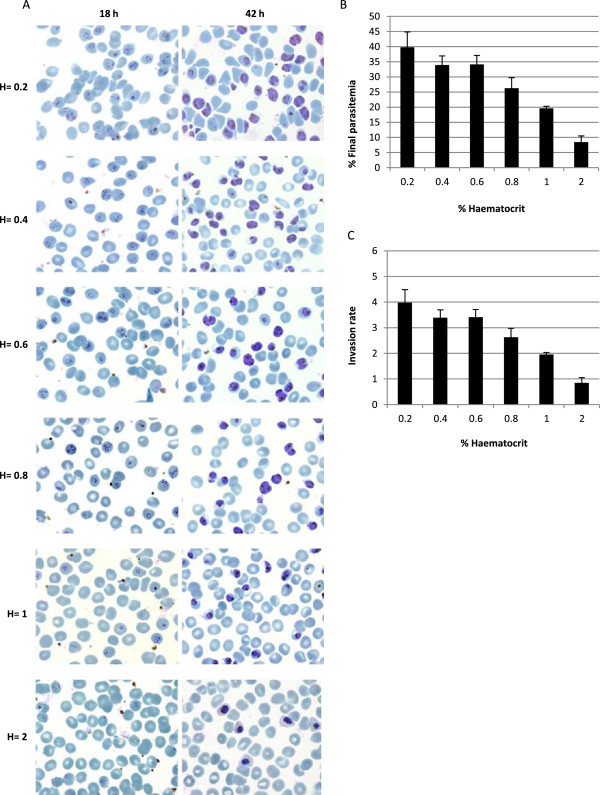
**Establishment of the haematocrit to obtain the highest invasion rate after magnetic columns purification. ***Plasmodium falciparum* iRBCs isolated with magnetic column were adjusted to 10% parasitaemia at different haematocrits (0.2, 0.4, 0.6, 0.8, 1 and 2). *Plasmodium falciparum* cultures were harvested after 18 and 42 hours to determine the parasitaemia reached in each condition. **A.** Giemsa-staining smears from cultures purified by magnetic purification and allowed to reinvade new uRBCs. **B.** Parasitaemia achieved at different haematocrits tested, estimated from blood smears (n = 3). **C.** Invasion rate calculated accounting the parasitaemia obtained after 18 hours (final parasitaemia) and the initial parasitaemia of the *P. falciparum* culture (10%) (final parasitaemia/initial parasitaemia). This rate indicates the capability of the parasites to invade new RBCs.

The influence of the haematocrit in the invasion capacity of parasites isolated by magnetic purification was also assessed. It is important to note that only fresh uRBCs were used in order to maximize invasion rates. A range of different haematocrits was tested while maintaining the parasitaemia at 10% in all the cultures. Isolated schizonts were kept in culture allowing them to invade new uRBCs under the different conditions described. Samples were collected after 18 hours in culture and parasitaemia levels were estimated by microscopy examination of Giemsa-stained smears (Figure 2A). Merozoites were able to infect new uRBCs, but the merozoite invasive capacity after schizont rupture was affected by the haematocrit level being lower at higher haematocrits (Figure 2A and B). The maximum invasion rate was 3.97 (± 0.50) and it was achieved when the haematocrit was fixed at 0.2% (Figure 2C). *Plasmodium falciparum* parasites purified by magnetic columns cultured at 1% haematocrit were able to reach a 19.58% (± 0.78%) parasitaemia (Figure 2B), a two-fold increase compared to *P. falciparum* cultures without any purification step (Figure 1A). Thus, the isolation of iRBCs using magnetic columns significantly increased the invasion rates as it was reported [29,30], a good indication of the viability of these parasites (Figure 2B and C). Moreover, additional sampling was made after 42 hours of incubation to verify the capability of the parasites to form healthy schizonts (Figure 2A). Parasites isolated using this protocol were able to complete their entire life cycle and kept the synchrony window obtained by performing three synchronization steps. When *P. falciparum* cultures with different haematocrits were compared a small number of gametocytes (around 1%) were observed at the highest haematocrits tested, revealing a stress condition typically generated by the parasite.

**Figure 3:**
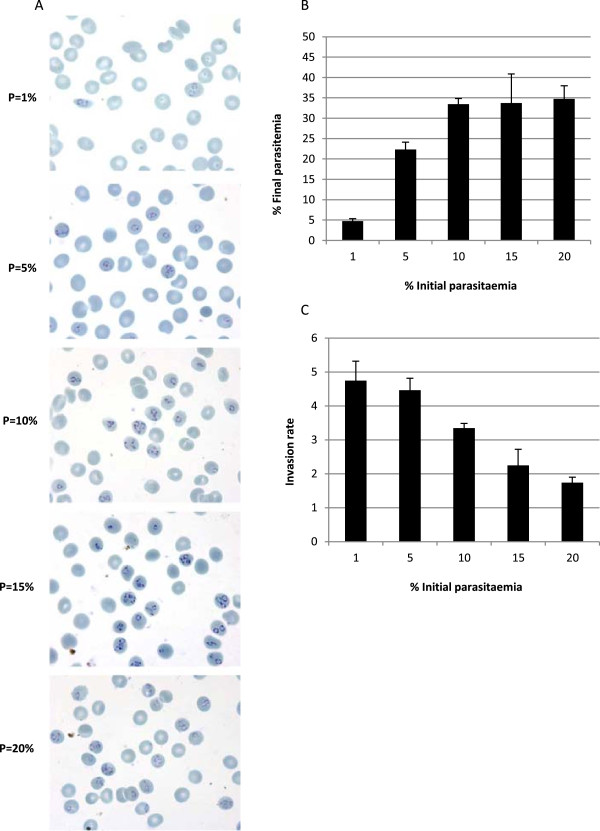
**Establishment of the initial parasitaemia after magnetic column purification. ***Plasmodium falciparum* iRBCs isolated with magnetic columns were adjusted to 0.2% haematocrit and different parasitaemias (1, 5, 10, 15, and 20%). *Plasmodium falciparum* cultures were harvested after 18 hours to determine the parasitaemia levels. **A.** Giemsa-staining smears from cultures purified by magnetic purification and allowed to reinvade new uRBCs. **B.** Parasitaemia achieved at 0.2% haematocrit with the different initial parasitaemias tested was estimated from blood-smears (n = 3). **C.** Invasion rate calculated accounting for the parasitaemia obtained after 18 hours (final parasitaemia) and the initial parasitaemia of the *P. falciparum* culture (final parasitaemia/initial parasitaemia). This rate indicates the capability of the parasites to invade new RBCs.

In order to assess the influence of the initial parasitaemia on the invasion rates and in the final parasitaemia achieved, a range of different parasitaemias were tested fixing a 0.2% haematocrit, which previously had been considered as the optimal. A maximum invasion rate of 4.74 ± 0.57 was obtained with the lowest initial parasitaemia tested (1%) (Figure 3C). However, with respect to the final parasitaemia reached, it was lower than that obtained with other conditions tested (Figure 3A, B and C). These results show that the parasite is able to invade new RBCs depending on the amount of parasites and the concentration of host cells present in the culture medium. This is consistent with previous studies in which the inhibition of the parasite development has been associated to the low pH due to the secretion of lactic acid (produced by the parasites) to the medium [8,9] and to the presence of auto-inducers that restrict the parasite propagation [10]. The isolation of the iRBCs using magnetic columns could be contributing to remove auto-inducers present in the media. The dilution of parasite cultures might also prevent parasite mechanisms involved in the restriction of parasite invasion. Importantly, removal of old uRBCs and addition of fresh uRBCs increases the invasion rates.

In order to check the reproducibility and robustness of the method previously described, eight independent replicates were used (Table 2). Final conditions of 0.2% haematocrit and 10% initial parasitaemia were able to achieve the highest parasitaemia with the minimum amount of initial *P. falciparum* culture. Forty per cent of parasitaemia was obtained after 18 hours in all performed experiments, showing the robustness of the method. Harvesting of high parasitaemia culture after 24 h or 48 h from the magnetic column purification is recommended as gametocytes appeared when cultures were maintained along the time, underlining the stress produced by the high parasite population.

**Table 2:** **Reproducibility of the final protocol using haematocrit 0.2 and initial parasitaemia of 10**%

**No**	**Final number of iRBCs**^**1**^	**Final parasitaemia (%)**^**2**^
1	9.63 × 10^7^	46.57
2	1.21 × 10^8^	39.42
3	8.50 × 10^7^	48.67
4	8.40 × 10^7^	39.17
5	1.10 × 10^8^	30.00
6	6.95 × 10^7^	39.78
7	8.33 × 10^7^	34.00
8	2.75 × 10^8^	33.46
**Average**	1.16 × 10^8^	38.88

^1^Number of iRBCs harvested 18h after the magnetic column purification, being initially adjusted to 0.2% haematocrit and 10% initial parasitaemia.

^2^Parasitaemia of the P. falciparum culture harvested after 18h from the magnetic column purification, being initially adjusted to 0.2% haematocrit and 10% initial parasitaemia.

Further studies were conducted to assess the susceptibility of the parasites to commercially available anti-malarial drugs, which affect different pathways of the parasite physiology (chloroquine, artemisinin, pyrimethamine, and atovaquone), to check that the viability of the parasite after magnetic column purification is not compromised. Similar IC_50_ and inhibition curve profiles for all the drugs tested were obtained for *P. falciparum* cultures isolated by the method previously described and for cultures not subjected to magnetic column purification (Table 3).

**Table 3:** **Effect of drugs on *Plasmodium falciparum *** **growth using cultures grown at low and high parasitaemia**

**IC50 (nM)**		
**Drug**	**P = 40%**	**P = 5%**
Atovaquone	1.00 ± 0.17	0.95 ± 0.14
Chloroquine	11.33 ± 3.48	16.30 ± 2.20
Artemisinin	40.75 ± 2.35	40.29 ± 6.05
Pyrimethamine	112.00 ± 53.62	117.59 ± 89.61

**Figure 4:**
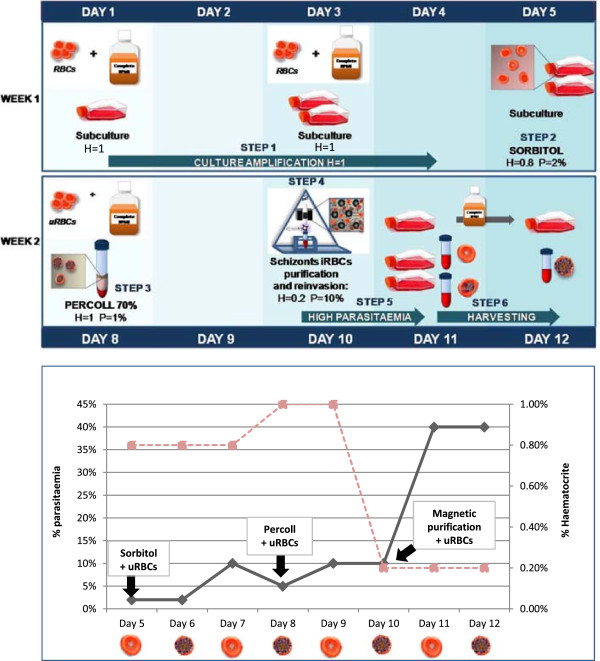
**Summary of the complete protocol. ***Plasmodium falciparum c*ulture is maintained at 1% haematocrit during the first week to scale up the *P. falciparum* culture. *Plasmodium falciparum* culture is synchronized with 5% wt/v sorbitol and then adjusted to H = 0.8% and P = 2%. Percoll is carried out after three days to purify the schizonts and *P. falciparum* culture is adjusted to H = 1% and P = 1%. Forty-eight hours later, *P. falciparum* culture enriched with mature forms is passed through the vario mac magnetic column to isolate the schizonts-enriched iRBCs. iRBCs obtained are adjusted to H = 0.2% and P = 10% and after 18 hours *P. falciparum ring-enriched* culture at approximately 40% parasitaemia is harvested. If a trophozoite or schizont-enriched population is required, media has to be changed and *P. falciparum* culture will be harvested next day.

An integrated view of the whole purification process is shown in Figure 4, where the scale up of the culture has been taken into account. If a large amount of culture is not required the procedure can be started at day 5. The applications can be numerous and the procedure can be partially or fully applied depending on the nature of the analysis required. For example, if a high synchrony is not essential, steps two and three can be omitted, or if only schizonts-enriched iRBCs are needed, the protocol could be stopped at step four after iRBCs purification, obtaining more than 98% parasitaemia.

This procedure could be applied to other *P. falciparum strains,* but the timing and the invasion conditions should be adjusted due to the different growth rates of each strain.

## Conclusions

A new procedure based on the use of sorbitol, Percoll and magnetic column purification is proposed to obtain *in vitro P. falciparum* cultures with a short-cycle window and with high parasitaemia levels (up to 40%) enriched at any intra-erythrocytic stage of the parasite. This method has several advantages over previously established methods: a) it is a less time-consuming protocol because it does not require continuous medium changes. Medium is only changed every two days and the same haematocrit is maintained until the magnetic column purification step; b) this method does not require long periods of *in vitro* incubation; the whole process takes two weeks if a large amount of culture is required. However, final 40% parasitaemia is achieved only one day after the last synchronization step by magnetic column purification without compromising parasite viability. This fact could overcome the disadvantage of other methods where unhealthy parasites can be produced due to the requirement of the gradual increase of the parasitaemia during several days with the subsequent lost of synchrony; c) it is highly reproducible as the same results are obtained from many different experiments; d) different *P. falciparum* stages can be harvested and compared amongst themselves. In addition, the harvested iRBCs come from viable parasites that have been able to invade new uRBCs.

Schizont-enriched iRBCs purified (98% parasitaemia) and *P. falciparum* cultures obtained using the complete protocol can be used for biochemical and molecular analysis where the uRBCs interfere with the biological material coming from the parasite. Although the maximal parasitaemia achieved is around 40-50%, this could be enough for some studies. For example, it could be applied for MS/MS assays or the analysis of post-translational modifications where the minimal manipulations of parasites are required to avoid loss of modified proteins. The method could provide high quality material for this kind of studies and could be useful for identifying new drug targets in drug discovery, an increasing challenge for the majority of current anti-malarial discovery programmes mainly based on phenotypic screenings where the targets remain unknown.

## Abbreviations

RBCs: Red blood cells; uRBCs: Uninfected red blood cells; iRBCs: Infected red blood cells.

## Competing interests

The authors declare that they have no competing financial interests.

## Authors’ contributions

LMC designed, performed experiments and drafted the manuscript. MJLF conceived the study, supervised experiments and contributed to drafting the manuscript. LS contributed to drafting the manuscript. MSR supervised experiments and drafted the manuscript. All authors read and approved the final manuscript.
